# eHealth literacy in older patients with cardiovascular disease: latent profile analysis and associated factors

**DOI:** 10.3389/fpubh.2026.1883826

**Published:** 2026-08-27

**Authors:** Mengjie Xu, Qianqian Sun, Qiping Chen, Ying Xia

**Affiliations:** Department of Cardiology, Nanjing Drum Tower Hospital, Affiliated Hospital of Medical School, Nanjing University, Nanjing, Jiangsu, China

**Keywords:** associated factors, eHealth, health literacy, latent profile analysis, older patients with cardiovascular disease

## Abstract

**Objective:**

This study aimed to explore the potential latent categories of electronic health (eHealth) literacy among older patients with cardiovascular diseases and analyze the factors associated with eHealth literacy profiles.

**Methods:**

From November 1, 2025 to April 15, 2026, we employed the convenience sampling method to conduct a survey on a total of 459 patients from a tertiary A hospital in China. The demographic information questionnaire and the Electronic Health Literacy Scale were employed to collect the sociodemographic information and eHealth literacy. Latent profile analysis was utilized to identify the latent categories of eHealth literacy among older patients with cardiovascular diseases. Univariate analysis and multivariate logistic regression analysis were conducted to explore factors associated with latent eHealth literacy profiles among these patients.

**Results:**

The mean total e-HEALS score was (25.16 ± 5.59), with mean scores of (15.59 ± 3.75), (6.41 ± 1.52), and (3.16 ± 0.96) for application ability, judgment ability, and decision-making ability, respectively. Latent profile analysis identified three distinct categories: low (28.3%), medium (50.3%), and high eHealth literacy (21.4%), demonstrating significant heterogeneity. Multivariate logistic regression showed that higher education level, more frequent Internet use, higher frequency of health information inquiry, regular physical activity, and greater perceived social support were positively associated with medium and high literacy categories, whereas advanced age and higher comorbidity burden were negatively associated.

**Conclusion:**

Older patients with cardiovascular diseases exhibit heterogeneous and generally moderate-to-low eHealth literacy. Individual behaviors and social support factors were associated with different eHealth literacy profiles, highlighting the need for tailored, multidimensional interventions and the development of disease-specific assessment tools to improve digital health engagement and self-management in this population.

## 1 Background

Cardiovascular disease (CVD) remains one of the leading causes of morbidity and mortality globally, particularly among older adults. According to the World Health Organization (WHO), CVD accounted for nearly 17.9 million deaths in 2019, representing 32 % of global mortality, with heart attacks and strokes being the primary contributors (1, 2). As the global population ages, the burden of CVD is expected to grow exponentially, especially in countries like China, where the population aged 60 and above is projected to surpass 30% by 2035 (3). The increasing prevalence of CVD in older populations presents significant challenges for healthcare systems, not only in terms of clinical care but also in promoting effective self-management and prevention strategies (4, 5).

Older adults are particularly vulnerable to the effects of CVD, as the physiological decline associated with aging contributes to the progression of cardiovascular diseases (6, 7). Moreover, the clinical presentation of CVD in older patients often deviates from the classic symptoms, leading to diagnostic difficulties and the risk of misdiagnosis (8). Older adults are also more likely to suffer from comorbid conditions, complicating treatment decisions and raising concerns about the safety and effectiveness of polypharmacy (9). As a result, tailored approaches that address the unique healthcare needs of older CVD patients are crucial for improving outcomes and enhancing quality of life (10).

In the context of modern healthcare, Electronic health (eHealth) literacy (11) has become an essential skill for individuals, enabling them to navigate the vast array of health information available online and to make informed decisions regarding their care (12). eHealth literacy is defined as the ability to search for, comprehend, assess, and apply health information from electronic resources (13). It encompasses several dimensions, including computer and internet literacy, the ability to understand and evaluate health information, and the capacity to apply this information to make decisions that promote personal health (14).

For older patients with CVD, eHealth literacy plays a vital role in self-management, medication adherence, and preventive care (15). Given that many older adults are increasingly using digital tools for health management, such as telemedicine and online health resources, enhancing their eHealth literacy can significantly improve disease outcomes and empower them to take an active role in managing their condition (16). However, older adults often face challenges related to technological proficiency, cognitive decline, and limited access to digital tools, which may hinder their ability to fully benefit from these resources (17). Moreover, disparities in eHealth literacy, driven by factors such as education, socio-economic status, and access to technology, can exacerbate health inequities within this population (18, 19).

Latent Profile Analysis (LPA) (20) is a powerful statistical method that can be used to explore the heterogeneous nature of eHealth literacy among older adults with CVD. LPA is a model-based clustering technique that identifies subgroups within a population based on patterns of observed data. These subgroups, or latent profiles, are unobservable but can be inferred through the analysis of observed variables. By applying LPA to this context, we can uncover distinct patterns of eHealth literacy in older patients, providing insights into how different individuals engage with digital health resources and what factors associated with their capacity to effectively use these tools. The findings of this study will help identify specific groups of patients who may need targeted interventions to enhance their eHealth literacy (21).

The objective of this study is 3 fold:

1. To examine the latent profiles of eHealth literacy in older patients with cardiovascular diseases, identifying distinct subgroups based on their ability to use digital health resources.
2. To investigate the socio-demographic, clinical, and technology-related factors that associated with eHealth literacy in this population, providing a comprehensive understanding of the determinants of digital health engagement.
3. To identify priority populations and factors associated with different eHealth literacy profiles, thereby providing an empirical basis for the development of profile-based targeted interventions to improve eHealth literacy among older patients with cardiovascular diseases.

## 2 Methods

### 2.1 Study design and participants

This study employed a cross-sectional, descriptive exploratory design, adhering to the STROBE guidelines (22) for reporting cross-sectional studies. Participants were recruited from the cardiology departments of a tertiary A hospital in China through convenience sampling. Inclusion criteria for participation were as follows: (1) aged 60 years or older, based on the commonly used definition of older adults in Chinese gerontological research (23) and the international healthy aging framework (24); (2) diagnosed with cardiovascular disease (CVD), including coronary heart disease, hypertension, heart failure, myocardial infarction, arrhythmia, or other clinically diagnosed cardiovascular conditions; (3) able to understand the survey instructions and independently complete the questionnaire; and (4) willing to provide informed consent. Exclusion criteria included: (1) any severe cognitive or psychiatric conditions that could hinder participation in the study; and (2) participants with more than 20% missing data on the questionnaire.

Data collection occurred between November 1, 2025, and April 15, 2026. The surveys were distributed and collected by trained researchers within hospital wards. Prior to obtaining informed consent, the researchers explained the purpose and procedures of the study. Once consent was obtained, participants completed the questionnaires independently. The completed questionnaires were collected immediately, with researchers reviewing them on-site for any missing responses. If any items were incomplete, participants were asked to fill in the missing information. Questionnaires were considered invalid if they contained substantial missing data (>20%) or showed obvious irregular response patterns indicating unreliable completion, such as selecting the same response option across all items without variation. This study was reviewed and approved by the Ethics Committee of Nanjing Drum Tower Hospital (Approval No.2025-0928-02). Participation is completely voluntary.

### 2.2 Sample size estimation

According to the recommendation (21), a sample size of 300–500 participants is recommended for latent profile analysis (LPA). Considering a 20% attrition rate, the initial target sample size was set to 375–625 participants to ensure that at least 300 valid participants would remain for analysis. Ultimately, 477 questionnaires were collected, of which 18 were excluded according to the predefined quality control criteria, resulting in 459 valid participants included in the analysis. The effective response rate was 96.23%, which is within the recommended range and ensures sufficient statistical power for the latent profile analysis., which is within the recommended range and ensures sufficient statistical power for the latent profile analysis.

### 2.3 Instruments

#### 2.3.1 Demographic and sociological situation questionnaire

The questionnaire designed by the researchers after literature review and discussion was adopted, including 17 items: Gender, Age, Education level, Marital status, Payment method of medical expenses, Residence status, Types of Cardiovascular Diseases, Number of other comorbidities, Current types of concomitant medications, Smoking status, Drinking status, Internet access frequency, Frequency of health information inquiry, assessed using four self-reported categories (“Always,” “Often,” “Occasionally,” and “Never”), reflecting participants' usual frequency of seeking health-related information, whether participated in any health education activities, Regular physical exercise is performed, Income was categorized according to the annual income classification criteria derived from the National Bureau of Statistics of China: low income ( ≤ 22,702 CNY/year), medium income (22,703–55,586 CNY/year), and high income (>55,586 CNY/year), whether be willing to use mobile applications or telematics information systems to track health status.

#### 2.3.2 Electronic Health Literacy Scale (eHEALS)

Electronic health literacy was measured using the Electronic Health Literacy Scale (eHEALS), developed by Norman and Skinner (11) and translated into simplified Chinese by Shuaijun et al. (25) using Brislin's classical back-translation method. The scale contains eight items across three dimensions: application ability of online health information and services (items 1–5), judgement ability to distinguish relevant online information (items 6–7), and decision-making ability to make health-related decisions based on the information (item 8). Each item is scored on a 5-point Likert scale, with 1 indicating “very incompatible” and 5 indicating “very compatible.” The total score ranges from 8 to 40, with higher scores indicating higher electronic health literacy; a score of 32 or above is considered qualified (26). In this study, Cronbach's α for eHEALS was 0.874.

#### 2.3.3 Perceived Social Support Scale (PSSS)

Perceived social support was measured using the Perceived Social Support Scale (PSSS), developed by Zimet et al. (27) and Paykani et al. (28) and adapted into Chinese by Jiang et al. (29). The scale is designed to assess the extent of social support perceived by individuals from different sources. It contains three dimensions: family support, which evaluates perceived understanding, care, and support from family members; friend support, which evaluates perceived understanding, care, and support from friends; and other support, which evaluates perceived support from other important individuals such as teachers, classmates, and colleagues. The scale consists of 12 items, with no reverse-coded items. Items are scored on a 7-point Likert scale, ranging from “strongly disagree” to “strongly agree.” Scores for the three dimensions are calculated as follows: family support (items 3, 4, 8, 11), friend support (items 6, 7, 9, 12), and other support (items 1, 2, 5, 10). The total score is the sum of all items, ranging from 12 to 84, with higher scores indicating greater perceived social support. Scores of 12–36 indicate low support, 37–60 indicate moderate support, and 61–84 indicate high support. In this study, Cronbach's α for the scale was 0.949.

### 2.4 Data collection procedures

All investigators received standardized training from the research team. Eligible participants were approached during hospitalization and provided with detailed verbal and written explanations of the study's purpose and procedures. Upon obtaining written informed consent, participants were asked to complete the paper-based questionnaire independently in a quiet environment. Investigators were available to clarify questions if needed. Questionnaires were checked on-site for completeness and logical consistency. Eliminate the questionnaires that have regularly and repeatedly answered all options, and then eliminate them according to the standard that the overall missing value of the questionnaire is greater than 10% or the missing value of a single scale in a local area of the questionnaire is greater than 40%. Two researchers independently entered the data into a secure database using double-entry verification to ensure accuracy.

### 2.5 Data analysis

All data were checked by two researchers and analyzed using IBM SPSS Statistics version 26.0 (IBM Corp., Armonk, NY, USA) and Mplus version 8.0 (Muthén & Muthén, Los Angeles, CA, USA). Descriptive statistics were used to summarize participant characteristics. The distribution characteristics of continuous variables were assessed before statistical analysis. Normally distributed variables were presented as means ± SD, whereas non-normally distributed variables were presented as medians (IQR), and categorical variables were presented as frequencies and percentages. Latent profile analysis (LPA) was conducted in Mplus 8.0 to identify subgroups of electronic health literacy based on response patterns across the eight individual eHEALS items. Models were sequentially fitted from one to multiple classes, and fit was evaluated using AIC, BIC, aBIC, Entropy, LMR, and BLRT. Lower AIC, BIC, and aBIC indicate better fit, and Entropy values closer to 1 indicate clearer class separation. Significant LMR and BLRT values (*p* < 0.05) indicate that a k-class model fits better than a k−1 class model. Subsequently, single-factor analyses (ANOVA, Kruskal–Wallis, χ^2^, or Fisher's exact test as appropriate) were performed to explore associations between socio-demographic and health behavior variables and latent classes (21). To account for multiple testing in the univariate analyses, *P*-values from the omnibus tests were adjusted using the Benjamini–Hochberg false discovery rate procedure. Both unadjusted and adjusted *P*-values were reported, and an adjusted two-sided *P*-value of < 0.05 was considered statistically significant. Variables showing statistically significant differences in the univariate analyses, including education level, Internet use frequency, number of comorbidities, regular physical activity, age, and perceived social support, were subsequently included in the multinomial logistic regression analysis to identify factors independently associated with latent profile membership (30).

## 3 Results

### 3.1 Characteristics and electronic health literacy of older patients with cardiovascular disease in this study

A total of 459 older patients with cardiovascular diseases, aged 60 to 87 (67.62 ± 5.78) years old, had an eHEALS score of (25.16 ± 5.59). The score for the dimension of application ability of online health information and services was (15.59 ± 3.75), the score for the dimension of judgment ability to distinguish relevant online information was (6.41 ± 1.52), and the score for the dimension of decision-making ability to make health-related decisions based on the information was (3.16 ± 0.96). Other general information is shown in Table 1.

**Table 1:** General information of older patients with cardiovascular diseases (*n* = 459).

Variable	Category	*N* (%)
Gender	Male	306 (66.7)
	Female	153 (33.3)
Age (years)		67.62 (5.78)
Education level	Primary school or below	50 (10.9)
	Junior high school	151 (32.9)
	High school/technical secondary school	132 (28.8)
	Bachelor	71 (15.5)
	Above bachelor	55 (12.0)
Marital status	Unmarried	2 (0.4)
	Married	392 (85.4)
	Divorced	43 (9.4)
	Widowed	22 (4.8)
Payment method of medical expenses	Urban resident medical insurance	278 (60.6)
	New rural cooperative medical scheme	92 (20.0)
	Public health insurance	61 (13.3)
	Others	28 (6.1)
Residence status	Living alone	19 (4.1)
	Living with spouse	210 (45.8)
	Living with children	196 (42.7)
	Others	34 (7.4)
Types of cardiovascular diseases	Hypertension	120 (26.1)
	Coronary heart disease	106 (23.1)
	Heart failure	110 (24.0)
	Arrhythmia	109 (23.7)
	Structural heart disease	14 (3.1)
Number of other comorbidities	None	27 (5.9)
	One	329 (71.7)
	Two	81 (17.6)
	Three	22 (4.8)
Current types of concomitant medications	One	57 (12.4)
	Two	189 (41.2)
	Three or more	213 (46.4)
Internet access frequency	Always	119 (25.9)
	Often	124 (27.0)
	Occasionally	130 (28.3)
	Never	86 (18.7)
Smoking status	Never	181 (39.4)
	Current smoker	75 (16.3)
	Former smoker	203 (44.2)
Drinking status	Never	203 (44.2)
	Current drinker	110 (24.0)
	Former drinker	146 (31.8)
Frequency of health information inquiry	Always	115 (25.1)
	Often	134 (29.2)
	Occasionally	131 (28.5)
	Never	79 (17.2)
Participation in any health education activities	Yes	300 (65.4)
	No	159 (34.6)
Regular physical exercise	Yes	326 (71.0)
	No	133 (29.0)
Income	Low	49 (10.7)
	Medium	251 (54.7)
	High	159 (34.6)
Willingness to use mobile applications or telematics systems to track health status	Yes	388 (84.5)
	No	71 (15.5)

### 3.2 The latent profiles of eHealth literacy in older patients with cardiovascular diseases

The latent profile analysis was conducted using the mean scores of the eight eHEALS items from 459 older patients with cardiovascular disease as observed indicators, adopting a person-centered approach. Models with one to five classes were sequentially fitted, and the results are shown in Table 2. When the number of classes increased to three, AIC, BIC, and aBIC decreased progressively, and Entropy exceeded 0.800, indicating classification accuracy greater than 90%. Both the LMRT and BLRT tests were significant (*P* < 0.001), and all class probabilities were above 5%, demonstrating good model fit and representative classification. Therefore, the three-class model was selected as the optimal solution.

**Table 2:** Goodness-of-fit indices for the latent profile model of older patients with cardiovascular diseases (*n* = 459).

Models	LL	AIC	BIC	aBIC	Entropy	LMR	BLRT	Category probability
1	−5035.399	10102.798	10168.863	10118.084	–	–	–	–
2	−4444.413	8938.826	9042.053	8962.710	0.914	< 0.001	< 0.001	0.322/0.678
**3**	**−4242.579**	**8553.159**	**8693.547**	**8585.641**	**0.936**	**< 0.001**	**< 0.001**	**0.283/0.503/0.214**
4	−4193.372	8472.744	8650.293	8513.824	0.886	0.204	< 0.001	0.237/0.129/0.423/0.211
5	−4151.362	8406.723	8621.434	8456.401	0.880	0.398	< 0.001	0.142/0.111/0.124/0.216/0.407

AIC, akaike information criterion; BIC, bayesian information criterion; aBIC, sample-corrected bayesian information criterion; LMRT, ro-mendel-rubin corrected likelihood ratio test; BLRT, bootstrap-based likelihood ratio test. Bold values indicate the selected optimal latent profile model.

**Figure 1:**
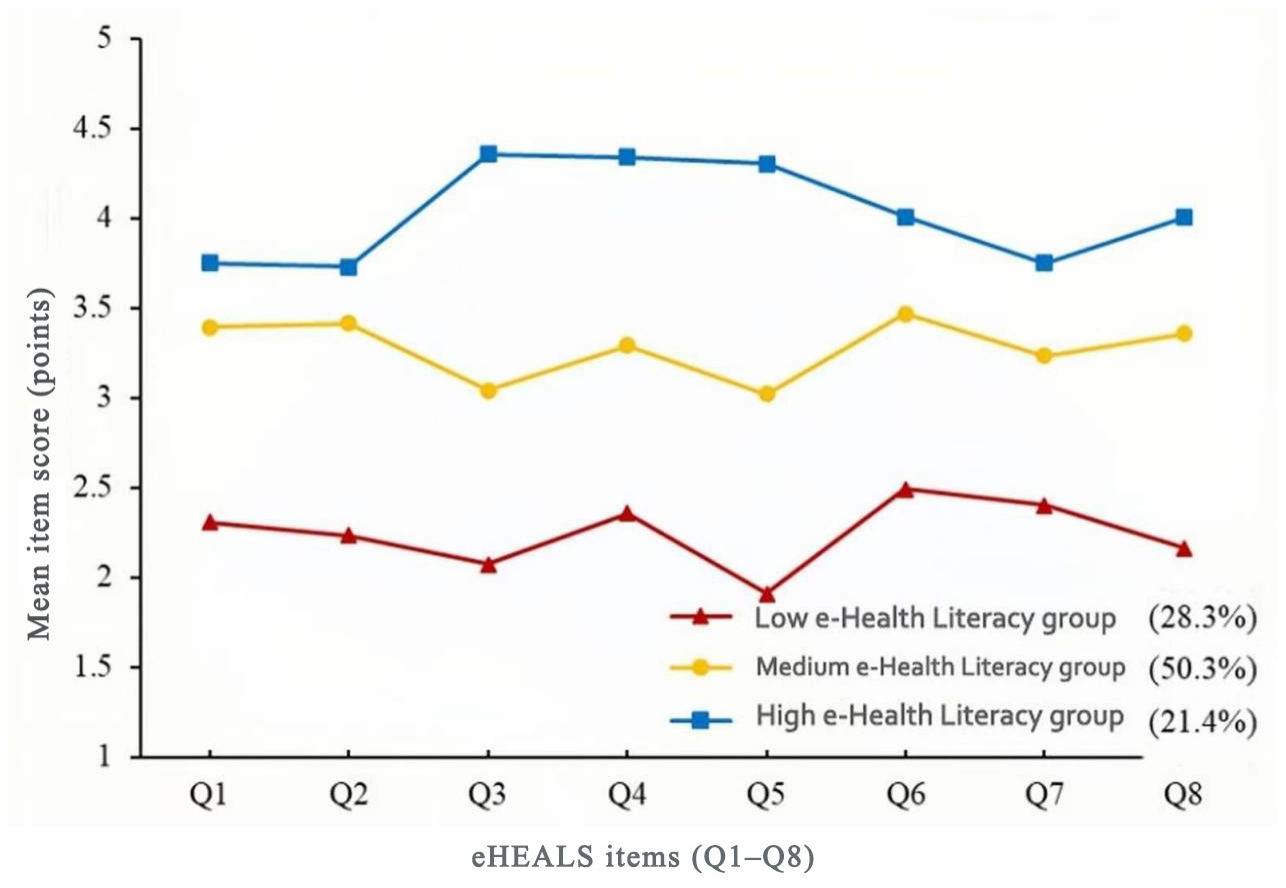
Characteristics of the latent profile distribution of eHealth literacy in older patients with cardiovascular diseases.

Based on the line plots of the eight eHEALS items, the three latent profiles were interpreted and named, as shown in Figure 1:

- Class 1 included 130 patients (28.32%), with all item scores lower than those of Classes 2 and 3, indicating weaker abilities in health information application, judgment, and decision-making. This profile was therefore labeled as the low electronic health literacy group.
- Class 2 included 231 patients (50.33%), with item scores between Classes 1 and 3, and certain items (e.g., decision-making ability) approaching the scores of Class 3. This profile was labeled as the medium electronic health literacy group.
- Class 3 included 98 patients (21.35%), with all item scores higher than Classes 1 and 2, reflecting stronger health information application, judgment, and decision-making abilities. This profile was labeled as the high electronic health literacy group.

### 3.3 Univariate analysis of factors associated with latent eHealth literacy profiles among older patients with cardiovascular diseases

The univariate analysis showed significant differences among the three eHealth literacy profiles in education level, number of comorbidities, Internet use frequency, regular physical activity, age, and perceived social support after correction for multiple testing (all adjusted *P* < 0.05; Table 3).

**Table 3:** Univariate analysis of factors associated with latent eHealth literacy profiles among older patients with cardiovascular diseases.

Variable	Low eHealth literacy (*n* = 130)	Medium eHealth literacy (*n* = 231)	High eHealth literacy (*n* = 98)	χ^2^/F	*P* (adjusted *P*)
Gender—Male	92 (70.8%)	147 (63.6%)	67 (68.4%)	2.067	0.356 (0.445)
Gender—Female	38 (29.2%)	84 (36.4%)	31 (31.6%)		
Education Level—Primary or below	16 (12.3%)	29 (12.6%)	5 (5.1%)	38.974	**< 0.001 (< 0.001)**
Junior high	46 (35.4%)	81 (35.1%)	24 (24.5%)		
High school/technical secondary school	40 (30.8%)	72 (31.2%)	20 (20.4%)		
Bachelor	21 (16.2%)	27 (11.7%)	23 (23.5%)		
Above bachelor	7 (5.4%)	22 (9.5%)	26 (26.5%)		
Marital status—Unmarried	1 (0.8%)	1 (0.4%)	0 (0.0%)	4.667	0.587 (0.652)
Married	110 (84.6%)	202 (87.4%)	80 (81.6%)		
Divorced	11 (8.5%)	21 (9.1%)	11 (11.2%)		
Widowed	8 (6.2%)	7 (3.0%)	7 (7.1%)		
Health insurance type—Urban	78 (60.0%)	142 (61.5%)	58 (59.2%)	2.625	0.854 (0.854)
New rural cooperative	24 (18.5%)	50 (21.6%)	18 (18.4%)		
Public	18 (13.8%)	28 (12.1%)	15 (15.3%)		
Others	10 (7.7%)	11 (4.8%)	7 (7.1%)		
Residency—Living alone	6 (4.6%)	8 (3.5%)	5 (5.1%)	8.674	0.193 (0.275)
With spouse	47 (36.2%)	114 (49.4%)	49 (50.0%)		
With children	64 (49.2%)	92 (39.8%)	40 (40.8%)		
Others	13 (10.0%)	17 (7.4%)	4 (4.1%)		
Number of comorbidities-−0	5 (3.8%)	5 (2.2%)	17 (17.3%)	31.155	**< 0.001 (< 0.001)**
1	96 (73.8%)	173 (74.9%)	60 (61.2%)		
2	21 (16.2%)	43 (18.6%)	17 (17.3%)		
3	8 (6.2%)	10 (4.3%)	4 (4.1%)		
Internet Use frequency—Always	25 (19.2%)	55 (23.8%)	39 (39.8%)	42.122	**< 0.001 (< 0.001)**
Often	20 (15.4%)	71 (30.7%)	33 (33.7%)		
Occasionally	47 (36.2%)	71 (30.7%)	12 (12.2%)		
Never	38 (29.2%)	34 (14.7%)	14 (14.3%)		
Physical activity—Yes	86 (66.2%)	159 (68.8%)	81 (82.7%)	8.478	**0.014 (0.024)**
No	44 (33.8%)	72 (31.2%)	17 (17.3%)		
Age (years, x ± s)	68.87 ± 5.94	67.81 ± 5.68	65.53 ± 5.25	9.941	**< 0.001 (< 0.001)**
Perceived social support (score, x ± s)	24.68 ± 7.24	27.18 ± 8.70	30.85 ± 11.01	13.477	**< 0.001 (< 0.001)**

Values outside parentheses are unadjusted *P*-values, and values in parentheses are *P*-values adjusted using the Benjamini–Hochberg false discovery rate procedure. An adjusted *P*-value of < 0.05 was considered statistically significant. Bold values indicate statistically significant results after Benjamini–Hochberg correction (adjusted *P* < 0.05).

### 3.4 Multinomial logistic regression analysis of factors associated with latent eHealth literacy profiles among older patients with cardiovascular diseases

Multiple logistic regression analysis was conducted to identify factors associated with the latent classes of electronic health literacy among older patients with cardiovascular disease, with the low eHealth literacy class as the reference group. Forward stepwise selection was applied (entry α = 0.05, exclusion α = 0.1), and the assignment of independent variables is shown in Table 4.

**Table 4:** Assignment of the independent variable.

Variable	Assignment
Education level	Primary or below = 1, Junior high = 2, High school/Technical secondary school = 3, Bachelor = 4, Above Bachelor = 5
Number of comorbidities	None = 0, One = 1, Two = 2, Three = 3
Internet use Frequency	Always = 1, Often = 2, Occasionally = 3, Never = 4
Frequency of health information inquiry	Always = 1, Often = 2, Occasionally = 3, Never = 4
Regular physical activity	Yes = 1, No = 2
Age	Entered as continuous variable
Perceived social support	Entered as continuous variable

The results of the regression analysis are presented in Table 5, indicating that education level, age, number of comorbidities, Internet use frequency of, perceived social support, frequency of health information inquiry, and Regular physical activity were significantly associated with latent class membership (all *P* < 0.05). Compared with the low eHealth literacy class, participants with higher education, more frequent Internet use, greater social support, more frequent health information inquiry, and regular exercise were more likely to belong to the medium or high eHealth literacy classes, whereas older participants and those with more comorbidities were more likely to remain in the low eHealth literacy class.

**Table 5:** Multinomial logistic regression analysis of factors associated with latent eHealth literacy profiles among older patients with cardiovascular diseases (*n* = 459).

Variable	Medium eHealth literacy					High eHealth literacy				
	β	*SE*	Wald *χ^2^*	*P*	*OR* (95%CI)	β	*SE*	Wald *χ^2^*	*P*	*OR* (95%CI)
Age	−0.037	0.021	3.301	0.069	0.963 (0.925, 1.003)	−0.150	0.032	21.633	**< 0.001**	0.861 (0.808, 0.917)
Perceived social support	0.042	0.014	8.750	**0.003**	1.043 (1.014, 1.073)	0.070	0.019	13.018	**< 0.001**	1.072 (1.032, 1.114)
Education level										
Primary or below	−0.636	0.568	1.254	0.263	0.530 (0.174, 1.611)	−2.288	0.785	8.487	**0.004**	0.101 (0.022, 0.473)
Junior high	−0.686	0.500	1.887	0.169	0.503 (0.189, 1.340)	−2.086	0.577	13.049	**< 0.001**	0.124 (0.040, 0.385)
High school/technical secondary school	−0.652	0.504	1.670	0.196	0.521 (0.194, 1.400)	−1.867	0.590	10.019	**0.002**	0.155 (0.049, 0.491)
Bachelor	−0.874	0.548	2.542	0.111	0.417 (0.142, 1.222)	−1.099	0.612	3.224	0.073	0.333 (0.100, 1.106)
Number of comorbidities										
None	−0.040	0.836	0.002	0.962	0.961 (0.187, 4.949)	1.979	0.986	4.031	**0.045**	7.238 (1.048, 49.969)
1	0.388	0.534	0.528	0.467	1.474 (0.518, 4.198)	−0.072	0.782	0.008	0.927	0.931 (0.201, 4.306)
2	0.475	0.582	0.667	0.414	1.608 (0.514, 5.030)	0.503	0.848	0.352	0.553	1.653 (0.314, 8.706)
Internet use frequency										
Always	0.921	0.352	6.839	**0.009**	2.513 (1.260, 5.012)	1.287	0.478	7.253	**0.007**	3.620 (1.419, 9.234)
Often	1.661	0.373	19.871	**< 0.001**	5.264 (2.536, 10.927)	1.856	0.513	13.095	**< 0.001**	6.401 (2.342, 17.493)
Occasionally	0.730	0.320	5.194	**0.023**	2.075 (1.108, 3.886)	−0.147	0.530	0.077	0.781	0.863 (0.305, 2.440)
Frequency of health information inquiry										
Always	0.998	0.384	6.742	**0.009**	2.712 (1.277, 5.759)	2.249	0.529	18.076	**< 0.001**	9.480 (3.361, 26.739)
Often	0.921	0.342	7.257	**0.007**	2.512 (1.285, 4.909)	1.373	0.507	7.338	**0.007**	3.946 (1.462, 10.655)
Occasionally	0.774	0.334	5.381	**0.020**	2.168 (1.127, 4.168)	0.390	0.536	0.528	0.467	1.476 (0.516, 4.223)
Regular physical activity										
Yes	0.079	0.255	0.097	0.756	1.083 (0.656, 1.786)	1.024	0.401	6.507	**0.011**	2.784 (1.268, 6.114)

Reference category: low eHealth literacy group. Bold values indicate statistically significant results (*P* < 0.05).

## 4 Discussion

### 4.1 There is significant heterogeneity in the electronic health literacy status among older patients with cardiovascular diseases.

The overall eHealth literacy of older patients with cardiovascular diseases in this study was relatively limited, with differences observed across application, judgment, and decision-making abilities. The overall level was broadly comparable with that reported among patients after percutaneous coronary intervention in Norway, but lower than that reported among some older adults with chronic diseases in China and patients with cardiovascular diseases in the United States (31, 32, 39). These differences should not be interpreted solely as variations in individual ability. Evidence from European settings suggests that digital health adoption among older adults is shaped by platform usability, digital self-efficacy, trust, previous technology experience, and access to clear information and professional support (33, 34). The healthcare context may also shape how older adults acquire and use digital health skills. In some European and North American healthcare systems, older adults may interact more independently with patient portals, remote monitoring systems, and primary-care digital services. By contrast, digital healthcare use among older Chinese patients is often embedded in hospital-based platforms and may involve greater assistance from family members (35, 36). Therefore, cross-country differences in eHealth literacy may reflect not only patients' individual capabilities, but also differences in digital infrastructure, healthcare delivery, platform design, professional support, and patterns of family involvement. These contextual differences should be considered when comparing findings across countries and when generalizing the present results beyond the Chinese healthcare setting. Latent profile analysis in our study identified three distinct categories of electronic health literacy: the low eHealth literacy category (28.32%), the medium eHealth literacy category (50.33%), and the high eHealth literacy category (21.35%), demonstrating significant heterogeneity. This three-class pattern is similar to the distributions reported in previous studies in the e- health literacy of older patients with chronic diseases (37–39), where comparable proportions of low, medium, and high literacy groups were observed. The replication of this latent structure across studies suggests that the heterogeneity in eHealth literacy is a consistent characteristic of older adult populations, reinforcing the importance of class-specific strategies to improve eHealth literacy. Although several eHealth literacy interventions have been implemented among patients with cardiovascular diseases, including structured training programs and digital tool–based initiatives such as remote rehabilitation portals, few have been tailored to the heterogeneous eHealth literacy profiles of older patients with cardiovascular diseases (40–42), these interventions have not yet been specifically designed to cardiovascular disease patients. Furthermore, the consistency of latent categories across populations suggests that these patterns may reflect intrinsic differences in engagement, cognitive, and experiential factors among older adults.

### 4.2 Factors associated with different eHealth literacy profiles among older patients with cardiovascular diseases

#### 4.2.1 Age

Within this older population, relatively advanced age was associated with a lower likelihood of belonging to the high eHealth literacy profile. Specifically, each 1-year increase in age was associated with lower odds of membership in the high profile (OR = 0.861, 95% CI 0.808–0.917), whereas no significant association was observed for the medium profile. This finding is consistent with previous research suggesting that age-related changes in sensory function, cognitive processing, memory, and confidence in technology use may create barriers to accessing, evaluating, and applying online health information (43, 44). Older patients may also have had fewer opportunities to acquire digital skills during their education and working lives, resulting in less familiarity with rapidly evolving digital platforms. These findings underscore the need for age-sensitive digital support, particularly for patients at the higher end of the age distribution (45, 46).

#### 4.2.2 Education level

Educational attainment was significantly associated with membership in the high eHealth literacy profile. Compared with patients with a bachelor's degree or above, those with lower educational attainment were less likely to belong to the high profile. This association was particularly pronounced among patients with primary school education or below (OR = 0.101, 95% CI 0.022–0.473). No significant association was observed between education level and membership in the medium profile. Formal education may contribute to eHealth literacy by strengthening reading comprehension, information processing, critical appraisal, and problem-solving abilities. Patients with higher educational attainment may also have greater previous exposure to computers, smartphones, and online information, enabling them to navigate digital health platforms more efficiently (47). In contrast, patients with lower educational levels may experience difficulties in accessing, evaluating, and applying online health information, which could limit their engagement with digital health resources (46). This pattern is consistent with prior research demonstrating that education plays a central role in shaping eHealth literacy (48, 49).

#### 4.2.3 Number of comorbidities

The number of comorbidities was associated with membership in the high eHealth literacy profile. Patients without additional comorbidities were more likely to belong to the high profile than patients with a greater comorbidity burden, whereas no significant association was observed for the medium profile. Multimorbidity increases the complexity of disease management by requiring patients to understand information concerning multiple conditions, medication regimens, dietary recommendations, symptom-monitoring requirements, and follow-up arrangements (50). Additional conditions such as diabetes and chronic kidney disease may also increase physical, cognitive, and psychological burdens, thereby reducing the time and resources available for engaging with digital health information (51, 52). Moreover, online information relating to multiple coexisting conditions may be fragmented or inconsistent, making it more difficult for patients to determine which recommendations are appropriate for their individual circumstances. Given the increasing burden of multimorbidity among older adults in China, the relationship between comorbidity complexity and digital health engagement warrants particular attention (53, 54).

#### 4.2.4 Regular physical activity

Regular physical activity was positively associated with high eHealth literacy among older cardiovascular patients. Compared with those who did not engage in regular exercise, patients who reported consistent physical activity were more likely to belong to the high literacy category (OR = 2.784, 95% CI 1.268–6.114), whereas no significant association was observed for the medium literacy group. This finding suggests that maintaining a routine of physical exercise may facilitate engagement with digital health resources (55). Regular physical activity has been shown to improve cognitive function, attention, and self-efficacy, which could enhance patients' ability to search for, evaluate, and apply online health information effectively (56). Furthermore, individuals who are physically active may exhibit higher health awareness and self-management motivation (57–59), making them more likely to seek and use eHealth resources to support their cardiovascular health. These observations underscore the potential synergistic relationship between healthy lifestyle behaviors and eHealth literacy, indicating that promoting physical activity may serve as a complementary strategy to improve digital health competence in older adults.

#### 4.2.5 Internet use frequency and frequency of health information inquiry

Frequent Internet use and health-information inquiry were both associated with more favorable eHealth literacy profiles, although their patterns of association differed. Frequent Internet use was associated with membership in both the medium and high eHealth literacy profiles. In particular, patients who reported using the Internet “often” had substantially higher odds of belonging to the high profile than those in the reference category (OR = 6.401, 95% CI 2.342–17.493). By contrast, frequent health-information inquiry was primarily associated with membership in the high profile, with patients who reported “always” seeking health information showing the strongest association (OR = 9.480, 95% CI 3.361–26.739).

These findings indicate that proactive information-seeking behaviors play a crucial role in shaping eHealth literacy. Older adults who routinely access the Internet and actively search for health information have greater exposure to diverse digital resources, are more familiar with navigating online platforms, and are better able to integrate relevant information into their health management routines (45). This behavioral engagement reflects both greater exposure to digital resources and more habitual interaction with digital health environments. Repeated Internet use and health-information seeking may provide older adults with more opportunities to develop familiarity with online platforms and practice locating, evaluating, and applying health information (60).

#### 4.2.6 Perceived social support

Higher perceived social support was associated with a greater likelihood of membership in both the medium and high eHealth literacy profiles. This finding is consistent with social support theory, which proposes that emotional, informational, and instrumental support can facilitate health-related behaviors and access to resources (61, 62). Patients with stronger perceived support may receive guidance, encouragement, or direct assistance in accessing and interpreting online health information, as well as motivation to apply this information to their self-care routines (63). In addition, social networks may provide exposure to diverse health information sources, opportunities for discussion, and feedback that reinforce effective use of eHealth platforms (64). The results indicate that social support may operate as an informational, behavioral, and motivational facilitator of eHealth literacy among older adults with cardiovascular diseases (65–67). Compared with the high eHealth literacy profile, fewer factors were significantly associated with membership in the medium profile. This difference may be related to the relatively smaller distinction between the medium and low profiles. Since the medium profile represents an intermediate level of eHealth literacy, some characteristics associated with higher literacy may not be sufficiently differentiated from those in the low profile, resulting in fewer significant associations.

### 4.3 Clinical implications and considerations for targeted intervention

The principal clinical implication of this study is that the identified eHealth literacy profiles provide an empirical basis for stratifying older patients with cardiovascular diseases according to their digital health support needs. The three latent profiles indicate that these patients differ not only in their overall level of eHealth literacy but also in their capacity to access, understand, evaluate, and apply digital health information. Therefore, the findings support a shift from uniform health education toward profile-based interventions in which the intensity and content of support are matched to patients' specific eHealth literacy characteristics (68).

The associated factors identified in this study further provide a basis for determining which patients should receive priority assessment and what forms of support may be required. Patients who were relatively older, had lower educational attainment, or had multiple comorbidities were less likely to belong to the high eHealth literacy profile, suggesting that these characteristics may be used as practical indicators for identifying patients who require earlier screening and more intensive assistance (69). In addition, less frequent Internet use, infrequent health-information seeking, and lower perceived social support were associated with less favorable eHealth literacy profiles. These findings suggest that targeted interventions should not be limited to teaching technical skills but should also strengthen opportunities for guided digital practice, health-information seeking and appraisal, and support from healthcare professionals and family members (70).

Based on this stratification, patients in the low eHealth literacy profile may require foundational digital-skills training, simplified educational materials, step-by-step demonstrations, and repeated supervised practice in using hospital applications and reliable cardiovascular health resources (71). Patients in the medium profile may benefit more from training focused on evaluating information credibility, recognizing misleading information, and applying digital information to medication management, symptom monitoring, lifestyle modification, and communication with healthcare professionals (72). Patients in the high profile may be offered more advanced digital self-management, remote monitoring, and decision-support resources (73). Thus, the present findings provide a preliminary framework for determining who should be prioritized, which abilities should be strengthened, and how intervention intensity may be differentiated across eHealth literacy profiles.

In clinical practice, brief eHealth literacy assessment could be incorporated into outpatient consultations, discharge education, cardiac rehabilitation, or routine follow-up to guide the allocation of digital health support (74). A blended approach combining face-to-face instruction with supervised digital practice may be particularly appropriate for patients with limited education, multiple comorbidities, or low confidence in technology use (75). However, because this study was cross-sectional, the identified associations should not be interpreted as causal effects. The proposed profile-based intervention framework therefore requires further evaluation in longitudinal studies and intervention trials.

## 5 Limitations

This study has several limitations. First, its cross-sectional design precludes causal inference regarding the factors associated with eHealth literacy. Second, participants were recruited from a tertiary A hospital in China, which may limit the generalizability of the findings to older adults receiving care in community, primary-care, or rural settings, as well as to healthcare systems with different levels of family involvement, digital infrastructure, and service delivery. Third, eHealth literacy and perceived social support were assessed using self-reported instruments and may not fully reflect participants' actual digital skills or available support. Future longitudinal and multicenter studies should include more diverse healthcare settings and additional factors, such as digital access, platform usability, and information quality, to further validate the identified profiles and associated factors.

## 6 Conclusion

In our study, older patients with cardiovascular diseases exhibit moderate-to-low and heterogeneous eHealth literacy, with three distinct latent categories identified. Education level, age, comorbidity burden, physical activity, Internet use, health information inquiry, and perceived social support were significantly associated with eHealth literacy levels. These findings highlight the need for tailored, multidimensional interventions and the development of disease-specific assessment tools to enhance digital health engagement and self-management in this population.

## Data Availability

The raw data supporting the conclusions of this article will be made available by the authors, without undue reservation.
